# Lupin Root Weevils (*Charagmus* spp., Curculionidae: Sitonini), a Lupin Pest: A Review of Their Distribution, Biology, and Challenges in Integrated Pest Management

**DOI:** 10.3390/insects12100950

**Published:** 2021-10-18

**Authors:** Diego Piedra-García, Christine Struck

**Affiliations:** Faculty of Agricultural and Environmental Sciences, University of Rostock, Satower Str. 48, 18059 Rostock, Germany; Christine.Struck@uni-rostock.de

**Keywords:** biological control, *Charagmus*, grain legumes, integrated pest management, *Lupinus*, pulses, *Sitona*, weevils

## Abstract

**Simple Summary:**

Lupin root weevils comprise two beetle species that cause major damage to lupin crops. These weevils have spread widely in Europe, but damage specifically occurs in very light sandy soils. The adults feed on the leaves of lupins; however, the larvae feed on the root systems, causing major damage. These larvae develop underground by feeding on the root nodules. Additionally, controlling the adults is challenging because of their cryptic behaviour. Conventional control with insecticides has limited success. Therefore, alternative management practices are needed. In addition to the biology of these pests, we provide an overview of several crop management measures as well as a range of biological control options. These measures could help control lupin pests, thus supporting the cultivation of lupins as a valuable crop rotation element and an important source of protein for food and feed.

**Abstract:**

Lupins (*Lupinus* spp.) are an ancient yet important legume crop. In Europe, the protein-rich seeds serve as livestock feed and have the potential to be a healthy vegetarian component of human diets. In some regions in north-eastern Europe, lupins are heavily damaged by two Curculionidae species, the lupin root weevils (LRWs) *Charagmus gressorius* (syn. *Sitona gressorius*) and *Ch. griseus* (syn. *S. griseus*). Narrow-leaved lupins (*L. angustifolius*) and white lupins (*L. albus*) are most affected. The weevils feed on lupin leaves, whereas their larvae feed on root nodules. Therefore, the larvae cause major root damage by creating lacerations that allow soil-borne plant pathogens to enter the plant tissue. These infestations lead to considerable yield losses and markedly reduced N-fixation of the root nodules. This review summarises the current knowledge on the origin, geographical distribution, and biology of these rarely described weevils. It focuses on management strategies, including preconceived insecticide use and potential ecological management methods, as key components of an integrated pest management programme against LRWs in Europe.

## 1. Introduction

The effort to increase the diversification of agriculture [1] together with the rising demand for non-genetically modified plant protein, has led to the growing interest in the cultivation of lupins in Europe. In the species-rich genus *Lupinus*, *L. angustifolius* (narrow-leafed lupin or blue lupin), *L. albus* (white lupin), and *L. luteus* (yellow lupin) are the most economically important *Lupinus* species in Europe [2,3]. Their seeds provide a substantial source of protein for livestock and humans. In addition, the plants are able to fix nitrogen and thus improve soil fertility. Therefore, lupins could be an important element in crop rotation [4]. Moreover, the new-world species *L. mutabilis* (subgenus *Platycarpos* (S. Watson) Kurl.) is receiving increasing attention as a biomass source for bioenergy proposes [5].

**Figure 1:**
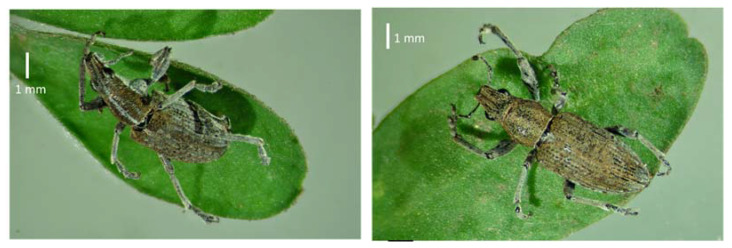
*Charagmus gressorius* (**left**) and *Charagmus griseus* (**right**).

In north-eastern Europe, successful lupin cultivation is severely curtailed by lupin-specific insect pests, namely two species of lupin-root weevils (LRWs), *Charagmus gressorius* (Fabricius, 1792) (syn. *Sitona gressorius*) and *Charagmus griseus* (Fabricius, 1775) (syn. *Sitona griseus*) (Coleoptera: Curculionidae), depicted in Figure 1 (right and left, respectively). 

Records of LRW-induced damage to lupins began in the 1930s with the increasing expansion of the newly bred ‘sweet’ low-alkaloid lupins varieties [6,7]. Both LRW species are native to the Mediterranean region, and historically, their main hosts were the old-world lupins (subgenus *Eulupinus* Aschers. et Graebn). Today, they are widespread throughout all of Europe after reaching Scandinavian and Baltic countries in the early 2010s [8,9]. Whereas Andersen [6,7] described LRWs as a new emerging pest in Central Europe, it can now be stated that they are settled residents. 

LRW adults feed on lupin leaves, causing typical U-shaped notches at the leaf margins. With an increasing number of individuals, the damaged area also grows. However, this herbivore foliar feeding behaviour has a negligible economic impact. More serious is the damage caused by the larvae feeding on the root nodules, causing considerable damage to the roots. As a result, the ability to fix nitrogen is disturbed, and the root lacerations serve as entry points for soil pathogens that infect the roots, affecting water and nutrient uptake.

Efficient management of weevils is difficult, and limited peer-reviewed research has been published about these pests. Therefore, we referred to comparative work with the better-studied, closely related *Sitona* species, which have very similar behaviour. However, the only detailed descriptions of the lifestyle of both LRWs to date were written by Andersen in the 1930s [6,7]. In our review, we continue the work of Andersen, adding the current knowledge about LRW distribution and key agricultural aspects with a focus on management strategies.

## 2. Systematics and Geographical Distribution

The species *Ch. griseus* was first described by J.F. Fabricius in 1775 [10] as *Curculio griseus* with habitats in Italy and England. In 1776, O.F. Müller [11] mentioned the occurrence of this species in Denmark and Norway. By contrast, *Ch. gressorius* (syn. *Curculio gressorius*) was originally specified in 1792 with its habitat in Italy and not able to spread north of the alps. Later, these species were assigned to the genus *Sitona* Germ. and the subgenus *Charagmus* Schoenherr, 1826 [12]. Recently, Velázquez de Castro et al. (2007) [12] clearly distinguished all genera of the tribe Sitonini from one another on the basis of morphological differences, and as part of this work, *Charagmus* was elevated to a genus. However, both *Ch. griseus* and *Ch. gressorius* are described as *Sitona* species in the literature.

**Figure 2:**
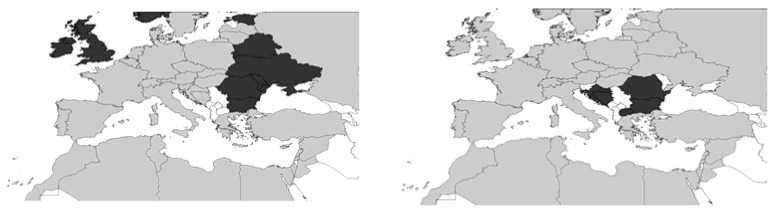
The geographical distribution of *Ch. gressorius* (**left**) and *Ch. griseus* (**right**) according to the database of Fauna Europaea [22,23]. Grey = present; black = absent, and white = no data.

In the late 19th century, *Ch. griseus* was already described as a pest in agriculture in Germany [13], and until the 1930s, this species was the only one found in Central Europe, whereas *Ch. gressorius* occurred south of the Alps [14,15]. Thus, *Ch. griseus* is the most historically widespread species in the West Palaearctic region, including Europe and North Africa (Morocco and Libya) [6,7,16] (Figure 2).

At present, *Ch. gressorius* is the most widespread LRW in the Central-Asiatic–European–Mediterranean region, including Turkey, Macaronesia, the Caucasus, Central Asia as far as Kazakhstan [16,17], and all Mediterranean countries [15,18]. Studies have demonstrated that this species spread from South Europe to Scandinavia [9] and the Baltic countries [8] (Figure 2) in the 20th century. The increased spread of the perennial garden lupin *L. polyphyllus* [19,20] may have facilitated the distribution of the weevils. These plants serve as the winter habitat for weevils and thus support their survival and dispersal in northern European latitudes. Dieckmann (1980) [15] posited that *Ch. gressorius* has a wide tolerance range for varying environmental factors. This species inhabits lowland areas as well as altitudes greater than 1000 m above sea level. This was confirmed by the work of Germann (2009) [21], who observed this species in Switzerland at 1200 m above sea level.

## 3. Biology and Ecology

### 3.1. Host Plants

All species of the six genera belonging to the tribe Sitonini feed on leguminous plants, mostly Fabaceae and some Mimosaceae. Species of the genus *Sitona* are well known worldwide as pests of numerous legume crops [12]. The adults of all Sitonini species feed on the green parts of their host plants, and their larvae feed on the root nodules. Both *Ch. gressorius* and *Ch. griseus* exclusively feed on species of Genisteae (genera *Cytisus*, *Genista,* and *Lupinus* [6,7,15] and Loteae (genus *Ornithopus*) [12]. The common broom (*Cytisus scoparius*, syn. *Sarothamnus scoparius*), a western Mediterranean plant, has spread throughout Europe through pastures and along roadsides as a perennial shrub. It also provides an ideal habitat for weevils for hibernation. Similarly, the spread of the New World perennial ornamental plant *Lupinus polyphyllus* in Europe may have contributed to the expansion of LRWs. A sample collected in Kaliningrad in 2010 was directly related to this invasive lupin species [24], and in Germany, larvae of *Ch. gressorius* were collected from the roots of *L. polyphyllus* [25].

Of the three Old World *Lupinus* species, *L. albus* has been cultivated in the Mediterranean countries for more than 3000 years, and *L. angustifolius* and *L. luteus* have been grown in Central Europe since the 19th century. After their successful breeding by Reinhold v. Sengbusch in the first half of the 20th century [26], the new ‘sweet’, low-alkaloid lupin cultivars (with less than 0.05% alkaloid content in seeds) of *L. albus*, *L. angustifolius*, and *L. luteus* were cultivated in different parts of the world. Today, the maximum alkaloid content in seeds is 0.05% for animal feed and 0.02% for human nutrition [2]. In the north-eastern parts of Germany and Poland, the cultivation of lupins is most widespread on light sandy soils. *Ch. griseus* feeding on the leaves of lupins in this region was first described at the end of the 19th century. *Ch. gressorius* first appeared as a new species in the 1930s [6].

A common characteristic of all host plants of LRWs is that they synthesize secondary metabolites of the toxic quinolizidine alkaloids (QAs) within their leaves [27], which are assumed to protect the plants against herbivores. A few insect specialists have evolved adaptations to tolerate QAs. One example is the lupin aphid *Macrosiphum albifrons* (Essig, 1911) [28]. This aphid species feeds on high-alkaloid lupins, whereas other aphid species (e.g., *Acyrthosiphon pisum* (Harris, 1776) and *Aphis fabae* (Scopoli, 1763)) avoid alkaloid-rich lupin cultivars but feed on sweet lupins with low alkaloid content [28]. In a 3-year field experiment with ‘bitter’ and ‘sweet’ genotypes of *L. albus*, *L. angustifolius*, *L. luteus*, and *L. nanus*, the LRWs *Ch. griseus* and *Ch. gressorius* did not exhibit clear differences concerning their feeding behaviour on leaves; the mean number of feeding notches varied from year to year. However, the bitter, high-alkaloid variety Azuro had the highest amount of feeding for all 3 years [29]. Furthermore, no correlation was observed between the leaf alkaloid content and the number of feeding notches on the leaves of different lupin genotypes. In conclusion, these experiments demonstrate that LRWs have adapted to the alkaloid spectrum of their specific host plants.

A second common characteristic of the LRW host plants is their ability to fix atmospheric nitrogen via symbiotic bacteria of the genus *Bradyrhizobium* Clade II (according to nodA phylogenetic analysis) [30,31]. *Bradyrhizobium* strains display a broad ecological spectrum and are thus widespread, forming symbioses with numerous legume genera [32]. Interactions between weevil larvae, their host plants, and the symbiotic bacteria are complex, and the relevant factor through which subterranean interaction is determined is not well-understood. Some research has been conducted with *Sitona* spp. Whereas several *Sitona* species feed exclusively on root nodules, others feed within the nodules only in juvenile stages and later feed directly on the root (reviewed by Johnson and Rasmann, 2015) [33]. It was shown that larvae of the clover root weevil *Sitona lepidus* are attracted by formononetin, a metabolite found in root nodules that actively fix nitrogen but not in non-active nodules [34]. In addition, female adults of *S. lepidus* prefer to oviposit on white clover plants with root nodules rather than on those without root nodules [35]. Although the *S. lepidus*–clover interaction can be understood as a model example, it should not be generalized and directly applied to the *Charagmus* species addressed here.

### 3.2. Damage

In Germany, the damage caused by LRWs remained minor and inconspicuous for many years. However, at the beginning of the 2000s, the cultivation of the widely grown yellow lupins had come to a standstill because of the disease anthracnose. Thus, the narrow-leafed lupins were increasingly cultivated instead, and the LRW infestation, along with feeding damage, became more and more severe. Results of field experiments in the northern German federal state of Mecklenburg-Western Pomerania revealed that the grain yield of *L. angustifolius* was reduced by up to 40% [36]. However, although the damage that LRWs cause in lupin fields is increasing annually, no specific research on its economic impact has been carried out to date.

**Figure 3:**
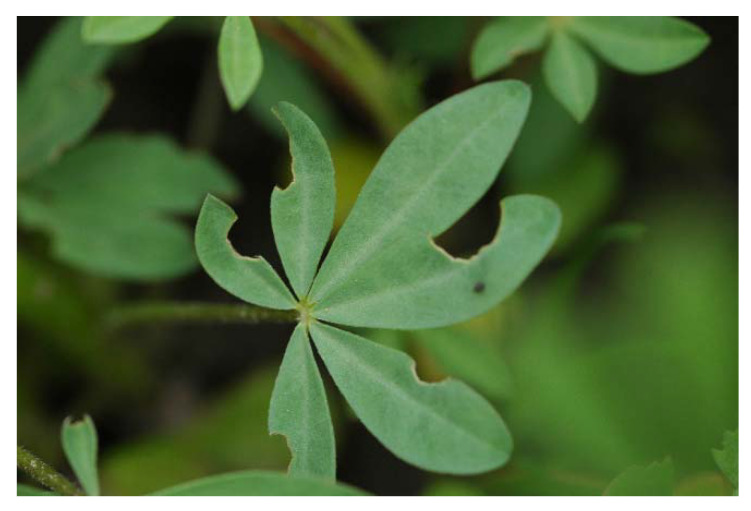
Lupin leaf U-shaped feeding notches.

Adult LRWs infest lupin fields in the spring and begin feeding on lupin leaf margins, causing a U-shaped feeding notch (Figure 3). In most cases, the damage is inconspicuous and appears to be economically negligible; however, in very early plant stages (seedling to six-leaf stages), this feeding can cause severe damage to narrow-leafed lupin stands [29]. Lohaus and Vidal (2010) [37] confirmed that even low *Sitona lineatus* densities of 0.2 adults per pea plant (20 individuals per m^2^) can cause considerable yield losses of seeds (18%) and pods (15%) per plant.

**Figure 4:**
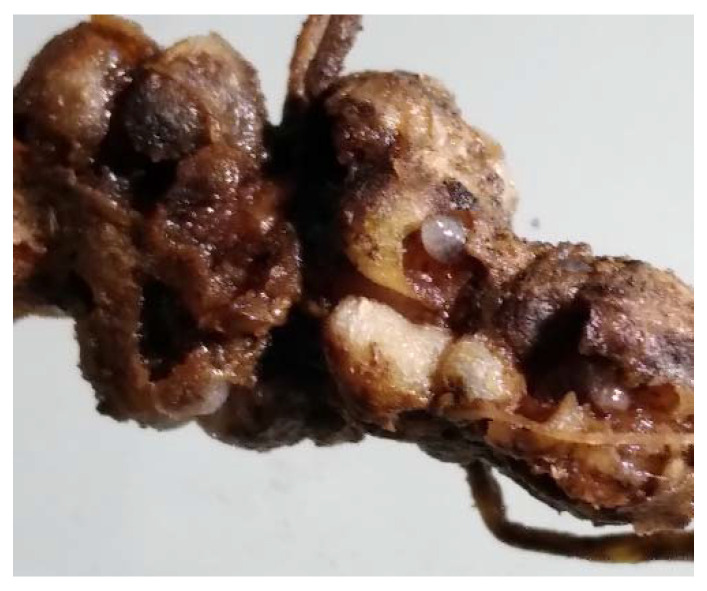
Lupin root nodules damage.

The soil-dwelling LRW larvae penetrate and feed on the lupin root nodules (Figure 4). In the worst cases, the lupin plants lose their complete root nodules and their ability to fix nitrogen. To a certain extent, the plant can compensate for the damage. This was also demonstrated in *Medicago sativa* plants, whose root nodules were eaten by *Sitona hispidulus* larvae [38]. However, the root damage caused by LRWs has a larger impact on lupin grain yields than the leaf consumption of the adult weevils [36]. The same observation was reported for *S. lineatus* feeding in pea fields [37,39].

Non-negligible losses occur because of secondary damage by soil pathogens, which use the feeding sites of larvae as entry ports into the root tissue. In addition, it was hypothesised that the larvae of other weevils, such as *Sitona hispidulus* (Fabricius, 1777), could be pathogen vectors because numerous pathogenic fungi were isolated from larval head capsules [40]. The damage caused by larval feeding is the opening for soil plant pathogens, such as *Fusarium* spp., *Phoma* spp., *Pythium* spp., *Rhizoctonia* spp., *Sclerotinia* spp., and *Thielaviopsis basicola* [41,42]. Hatcher (1995) [43] also outlined this connection in a review. These pathogen infections lead to a reduction in water and nutrient supply. A detailed study revealed that the reduction in overall nodulation in peas was stronger when the roots were exposed to both the pea leaf weevil larvae and *Fusarium avenaceum*. In addition, the authors observed that more weevil larvae survived and pupated when they consumed the root nodules of infected plants as opposed to healthy ones, indicating a mutual interaction between the pea leaf weevils and *F. avenaceum* [44].

Another issue that should be considered is nitrogen (N) fixation. The cultivation of grain legumes plays an important role in organic farming, especially on farms without livestock, because nitrogen availability is low. In comparison with other grain legumes, lupins fix high amounts of nitrogen; some species fix up to 527 kg N · ha^−1^ [5]. However, this largely depends on bioclimatic and soil conditions [45,46,47]. Nevertheless, little research has focused on the possible losses of nitrogen fixation caused by weevil larvae damage to root nodules. Losses in nitrogen fixation due to larval feeding processes of LRWs on lupin root nodules have not been determined. Corre-Hellou and Crozat (2005) [48] reported an influence of pea weevils together with high weed pressure on the N fixation performance of peas. The damage caused by *Sitona flavescens* (Marscham, 1802) on *Trifolium repens* led to a 42% nitrogen reduction in the plants compared with control plants [49], and *S. hispidulus* infestation in white clover can lead to nitrogen stress [40].

### 3.3. Life Stages

The morphology of *Ch. gressorius* and *Ch. griseus* is described in detail by the authors of [6,7], who also characterized the very similar life cycles of the two species. 

**Figure 5:**
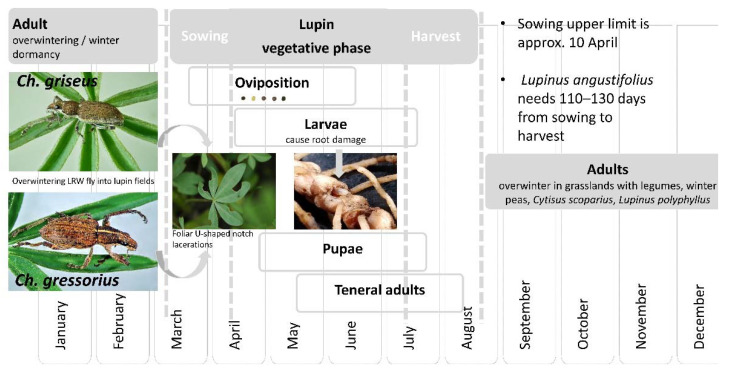
Generalized life cycle of lupin root weevils (LRWs) in Central Europe.

LRWs appear in fields from overwintering sites to feed on leaves when lupin seedlings emerge (Figure 5) [50]. Once the weevils have appeared, they move on the soil surface or the plants and start mating, and the females lay yellowish-white, ellipsoid eggs, which turn dark after 2–3 days. They are scattered on the plants and the soil surface. The eggs of *Ch. griseus* are larger on average (length 0.895 mm, width 0.664 mm) than those of *Ch. gressorius* (length 0.558 mm, width < 0.526 mm). This fact may influence the habitat of the beetles because *Ch. griseus* is found predominantly on light and very sandy soils [15], where their eggs can be washed into the large-pored soil by rainwater. *Ch. gressorius* is also often found on areas with sandy loam [15], into which the smaller eggs can be washed into the soil by rainwater.

Larvae of both LRW species are very similar in colour and shape. Typically, they are white to pale yellow in colour, are leg- and eyeless, have a light yellow to brown head capsule, and are grub-shaped with setulae (bristles) [7]. A recent publication provided a deep anatomical larval description of mature larvae and pupae of *Ch. gressorius* using scanning electron microscopes [25]. To our knowledge, no published studies have investigated the number of instars of LRWs. Related Sitonini species have four instars (*S. cylindricollis*, *S. scissifrons*, and *S. regensteinensis*) or five instars (*S. discoideus*, *S. lineatus*, and *S. lividipes*) [51,52]. Only one pupal description of *Ch. gressorius* (7.5–9.4 mm length, 4.3–5.0 mm width, 1.3–1.5 mm head width at the level of the eyes) has been published [25]. The literature includes fewer descriptions of Sitonini species in the pupal stage, and thus no identification keys are available [25]. The pupae of both species have white bodies. It is clear that more studies are needed regarding pupal identification, sexing, and eclosion.

### 3.4. Phenology

According to Dieckmann’s classification [15], three *Sitona* weevil development types are known in Central and Northern Europe: *lineatus*, *humeralis*, and *flavescens*. The *lineatus* type is the most common; the adults lay eggs in the spring, and the new generation appears in the summer and overwinters. Both the *humeralis* and the *flavescens* development types lay eggs in the late summer and autumn. The humeralis type eggs overwinter, and in the *flavescens* type, the larvae hatch in autumn and overwinter. 

Dieckmann [14] classified *Ch. griseus* and *Ch. gressorius* as members of the *flavescens* development type (although the classification of the latter is somewhat unclear), laying eggs in summer and autumn, and they thus have an overwintering larval stage in addition to the overwintering adults. Gosik and Sprick [25] found larvae and pupae of *Ch. gressorius* in late June and July under *L. polyphyllus*, but they found no larvae in April and October; therefore, they questioned the *flavescens* development type for this species. However, in agricultural practice, lupins are typically grown in a crop rotation. In this case, the beetles always migrate into the newly cultivated fields during the spring, where larvae of the previous year do not occur. 

**Figure 6:**
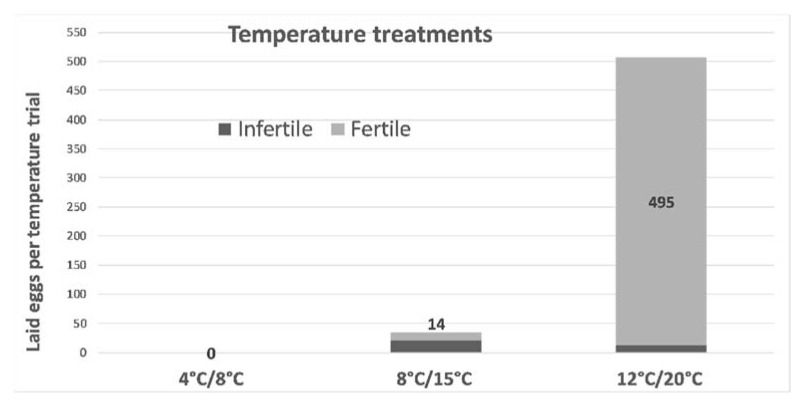
Oviposition of *Ch. gressorius* at three different day/night temperature treatments (N = 120 individuals; a significant difference was observed between treatments, Wilcoxon rank-sum test, *p* < 0.05; Lüdtke and Struck, unpublished data).

To obtain more information about the oviposition conditions, we performed laboratory experiments. In total, 120 *Ch. gressorius* individuals were collected after the lupin harvest in August 2017 and kept separately for males and females at 4 °C in darkness. The experiment began on 25 October 2017 and lasted 56 days. In experiments performed directly after collecting the beetles in August, mating and oviposition did not occur. Therefore, we assumed that the beetles require a dormant phase. In the experiment setup, the temperature was tested as the influence factor for the oviposition behaviour and egg fertility. The trial was performed in 12 perforated plastic boxes, each with six female and four male individuals. Three different temperatures regimes were examined in parallel: 4 °C/8 °C, 8 °C/15 °C, and 12 °C/20 °C corresponding to night and day temperatures. The eggs were collected every 2 or 3 days and transferred to Petri dishes with 1.5% water agar. The egg fertility was tested by counting the number of hatched larvae. The results revealed that at night/day temperatures of 4 °C/8 °C, no eggs were laid. At the 15 °C day temperature, 35 eggs were laid in total, of which 14 were fertile. The sequence at 20 °C day temperature must be highlighted because 507 eggs were laid in total, and just 12 of them were not fertile. This study also determined that temperature plays a role in the incubation time after oviposition. In the 15 °C day temperature trial regime, the eggs required an average of 26.2 days to hatch. In the 20 °C regime, the hatching time was reduced to 14.5 days (Figure 6). In brief, temperature has a strong influence on the LRW population, as it does on other Sitonini species.

## 4. Integrated Pest Management

*Sitona* weevils are found in a wide variety of crops worldwide, and accordingly, the literature on the management of these pests is abundant. By contrast, few sources have been published for LRWs, as large populations of these species are only found in lupin fields in a relatively small region of Central Europe. Andersen (1937) [6], who was the first to describe lupin weevils as pests in lupins, did not provide any information on the need for their control.

### 4.1. Monitoring

The timely monitoring of pests is a basic requirement for pest control. Direct monitoring of LRWs is extremely difficult because the adults are rarely seen. When attacked or physically provoked, they drop to the soil and exhibit a death-feigning (thanatosis or tonic immobility) reflex, a phenomenon that is taxonomically known as an anti-predatory defence mechanism [53]. Therefore, a control decision cannot be based on sampled weevils. However, the monitoring of adult LRWs is possible through soil-based traps, such as pitfall traps, because the beetles move on the soil after reaching the lupin field and rarely fly. This is very similar to the pea weevil *S. lineatus*, which also crawls through the host plant field [54]. Therefore, pitfall traps are suitable for monitoring the initiation of LRW feeding activity in the spring.

To make control decisions, it is useful to record the number of U-shaped feeding notches on the leaves, rather than weevil population densities [36]. In this manner, the larval damage can be estimated using the correlation between the spring feeding activity and the subsequent larval population [55].

### 4.2. Chemical Control

To gather stronger knowledge about the extent of weevil damage and its impact on the *L. angustifolius* grain yield, control measures were carried out with chemical insecticides. Treatments persisted in seed coating with weekly neonicotinoid and insecticide spraying using pyrethroids from emergence until the formation of flower buds. The results demonstrated that the grain yield in insecticide-treated lupin crops was up to 40% higher than that of untreated plots [36]. However, this extreme treatment was only feasible for experimental purposes. In Germany, according to Bundesamt für Verbraucherschutz und Lebensmittelsicherheit (BVL) 2021 [56], seed treatments with neonicotinoid insecticides are not currently allowed, and insecticide applications in legumes are only allowed twice per season. Matyjaszczyk (2020) [57] highlighted that different active substances are used in eight studied EU countries (Austria, Belgium, Czech Republic, Germany, Holland, Hungary, Poland, and Slovakia). The accreditations are unique: maltodextrin in Germany, chlorpyrifos in Hungary, thiacloprid in Poland, and three substances in Holland: pirimicarb, *Bacillus thuringiensis* subsp. *aizawai*, and *Metarhizium anisopliae* var. *anisopliae* strain BIPESCO 5/F52.

In other grain legumes, the most common foliar insecticide active ingredient against weevils is currently lambda-cyhalothrin [58]. However, adult weevil populations can be reduced using one or two insecticide treatments while not affecting yield loss. The proper timing of insecticide application is essential because it is useful only when the weevil infestation is beginning and before eggs are laid. This timing is quite early—during the four- to six-leaf stage [59]—and more LRWs may still fly in several days later, thus requiring further treatments. In conclusion, LRW control is limited through legal restrictions on the application of chemical insecticides.

### 4.3. Crop Management Measures as Control Options

Various potential management strategies can be considered as control options. Due to the limited experience with LRWs so far, it may be advisable to consider the results in the related *Sitona* species. In general, the diversification of crop rotation, including pulses, is widely recommended [1,4,60]. Crop rotation forces *S. lineatus* to undertake migratory flights in the spring. However, the crop rotation method is not sufficient to decimate the beetles significantly, as they are capable of migratory flights (Review Vankosky et al., 2009) [52].

A key component for the reduction of *S. lineatus* is tillage. In a comparison between conventional tillage and no-tillage (or direct-seeding system) pea cultivation, Hanavan et al. observed significantly fewer adults [61] and larvae [62] in conventional tillage plots. The authors of these studies assumed from similar investigations that a crucial factor for this reduction was the cooler temperature in the no-tillage plots. In addition, pea plants emerged earlier in conventional tillage plots and thus feeding and oviposition began earlier than in the no-tillage plots [61,62]. In this context, the sowing date also plays a role. If the pea leaves are available earlier, colonisation by pea weevils begins earlier [63]. The authors also demonstrated that cereal residues of the previous year’s crop inhibited the ability of the beetles to locate the host plants. The later sowing date, together with the crop residues, could make the no-tillage system a better crop management method compared with others.

Another option for pest control in integrated pest management is the use of intercropping, in which two or more crops are grown on one plot, thus representing a form of habitat management [64,65]. Mixtures of crop plant species increase the plant diversity and suppress the populations of specific insect pests. An intercropping field experiment was conducted in Poland with plots of narrow-leafed lupins alone or intercropped with spring triticale. The authors observed more *Charagmus* weevils in the pure lupin plots than with intercropping. Nevertheless, they did not detect any differences regarding the number of leaf feeding sites [66].

### 4.4. Host Plant Resistance

Host plant resistance against weevils may contribute to an effective management strategy involved in an integrated pest management (IPM) programme [67]. All species of the tribe Sitonini, including the genera *Charagmus* and *Sitona*, are specialists that feed on legumes. As such, they are highly adapted to their host plants or, in some cases, to closely related plant species. Single plant traits can affect such plant–insect interactions. For example, pea leaf weevils (*S. lineatus*) were strongly influenced by a reduction of the leaf surface wax of pea plants. Pea leaf weevils were three times more abundant in peas with reduced wax than in normal pea plants [68]. Various plant species were tested to identify differences between genotypes in terms of their attractiveness to herbivorous insects. Murray et al. (2007) [69] tested 16 red clover genotypes (*Trifolium pratense*) regarding the attractiveness of *S. lepidus* adults (syn. *S. obsoletus*) and their larvae. One of these genotypes was clearly preferred by adults and larvae. By contrast, in another clover genotype, the preference for a root did not coincide with the preference for leaves of the same genotype [69]. Considerable and highly significant differences between resistance against the root nodule feeding behaviour of *Sitona crinitus* on lentil (*Lens culinaris*) cultivars were reported [70].

Plant resistance traits as secondary metabolites or volatile compounds may alter the behaviour of insect pests and can thus be used in push–pull strategies [71]. Moreover, plant toxic compounds, such as the lupin alkaloids of the quinolizidine group, serve as defence chemicals against phytophagous insects [72]. In a 3-year field experiment with different *Lupinus* species and varieties, the toxic, high-alkaloid variety Azuro was the most favoured plant by the LRWs, showing the highest amount of leaf feeding damage for all 3 years [29] and indicating that *Charagmus* weevils are adapted to the lupin alkaloids.

### 4.5. Natural Enemies and Potential Biological Control

The abovementioned crop management measures are typically not fully effective against LRWs; therefore, biological control programmes using predators, parasitoids, and microbial control agents may provide more effective control options [73]. As the species of Curculionidae are predominantly herbivorous and many of them are major pests, they are often used as target organisms of biocontrol strategies [74]. The search for natural enemies has revealed that along with entomopathogens, insects could also be used as parasitoids or predators of *S. lepidus* [75] and *S. lineatus* [76,77].

The successful control of the alfalfa leaf weevil *Sitona discoideus* in Australia and New Zealand [78,79] and the clover root weevil *S. lepidus* (syn. *S. obsoletus*) in New Zealand was achieved in this manner [80]. In this study, the solitary wasp *Microctonus aethiopoides* Loan (Hymenoptera: Braconidae), which originated in North Africa and Europe, was introduced. These wasps have a wide range of natural hosts in the group of Curculionidae, including both *Ch. gressorius* and *Ch. griseus* [74,81]. However, no successful biological control measure has been established to date against these two species. Nevertheless, perspectives could be derived from other weevils with soil-dwelling larvae. For example, the eggs of *S. lineatus* were destroyed or removed by several ground beetle species living as egg predators [77] or egg parasitoids, such as the chalcid wasp *Anaphes diana* (Hymenoptera: Mymaridae) [76].

Research has addressed the potential of entomopathogenic fungi (EPFs) and nematodes (EPNs) [33]. However, it is challenging to identify fungal isolates that are effective against both larvae and adult weevils [82,83] and can survive under a given field soil environment [84]. Nevertheless, several commercially available EPF products have been successfully used against the below-ground larvae of the black vine weevil *Otiorhynchus sulcatus* [85,86].

The first trials with EPNs have also shown promising results. In laboratory experiments, high mortality of *Sitona lineatus* adults after exposure to *Steinernema carpocapsae* was observed [87]. Another work reported that *Heterorhabditis bacteriophora* (strain Oswego) is able to inhibit the development of late *Sitona hispidulus* instars [88]. The efficacy of EPNs is often restricted by environmental conditions [89]. When temperature and soil type are optimal, EPN application in the field can be a successful measure against soil-dwelling larvae [90].

In conclusion, numerous studies have indicated that in fields, predators, parasitoids, and soil-dwelling pathogens have the potential to be effective components of biocontrol of Curculionidae larvae by considerably reducing weevil pests. No previous studies carried out with both chemical insecticides and biological agents observed fully successful results. The legal restrictions in the EU and the ecological situation do not allow further chemical control measures. Therefore, future work must focus on improved biological control.

## 5. Conclusions

LWRs are a continuing problem for European lupin cultivation, especially for organic cultivation, because larval feeding on the roots reduces N fixation. Above ground, the damage is not very conspicuous, and it is thus often underestimated by farmers. As with many root-damaging pests, management is challenging. However, although LRW-induced damage in lupin fields is increasing annually, no specific research on economic impact has been carried out. Chemical insecticides used against adult weevils do not provide effective control. Research on closely related weevil species has provided control options. However, the development of novel, improved approaches is needed, including a decision support model. This requires in-depth studies of the developmental biology of the weevils and, above all, species-specific knowledge of both *Ch. griseus* and *Ch. gressorius*.

## Data Availability

Not applicable.
